# Editorial: New trends in vascular biology 2024

**DOI:** 10.3389/fcvm.2025.1649558

**Published:** 2025-07-24

**Authors:** Sarvesh Chelvanambi, Hong Chen, Margreet R. De Vries, Yun Fang, Gabrielle Fredman, Delphine Gomez, Hiroshi Iwata, Shizuka Uchida, Hiromi Yanagisawa, Masanori Aikawa

**Affiliations:** ^1^ Center for Interdisciplinary Cardiovascular Sciences, Brigham and Women’s Hospital and Harvard Medical School, Boston, MA, United States; ^2^ Vascular Biology Program, Department of Surgery, Boston Children’s Hospital and Harvard Medical School, Boston, MA, United States; ^3^ Einthoven Laboratory for Experimental Vascular Medicine and Regenerative Medicine, Leiden University Medical Center, Leiden, Netherlands; ^4^ Department of Medicine, Biological Sciences Division, The University of Chicago, Chicago, IL, United States; ^5^ Department of Molecular and Cellular Physiology, Albany Medical College, Albany, NY, United States; ^6^ Department of Medicine, Division of Cardiology, University of Pittsburgh, Pittsburgh, PA, United States; ^7^ Department of Cardiovascular Biology and Medicine, Juntendo University Graduate School of Medicine, Tokyo, Japan; ^8^ Center for RNA Medicine, Department of Clinical Medicine, Aalborg University, Copenhagen, Denmark; ^9^ Life Science Center for Survival Dynamics, Tsukuba Advanced Research Alliance, University of Tsukuba, Tsukuba, Japan

**Keywords:** vascular disease, cardiometabolic disease, macrophage, PCSK9, vascular imaging, endothelial-mesenchymal transition, COVID-19, data science

**Editorial on the Research Topic**
New trends in vascular biology 2024

Despite the development of potent drugs for modifiable risk factors, such as statins, and advances in mechanistic biomedical research, vascular disease remains the No.1 killer globally and represents a huge cost to public health (1–3). The underlying mechanisms remain incompletely understood and effective new therapies are needed (4). Such major challenges have promoted technological innovations and their implementations in vascular research (5). Unmet clinical needs and exponential technological development have synergistically advanced vascular medicine. Addressing the challenges associated with the complexity in treating cardiovascular disease requires integration of cross-disciplinary approaches and knowledge (6).This Research Topic thus reports new trends in a wide range of vascular medicine research from fundamental basic science to translational medicine to clinical studies.

This collection particularly focuses on four different areas in vascular medicine; (i) new understandings of cardiometabolic vascular dysfunction; (ii) novel imaging approaches; (iii) COVID-19 pandemic; and (iv) data science.

## New understandings of cardiometabolic vascular dysfunction

In the first area, we featured four articles which identified novel contributors of cardiometabolic vascular dysfunction. Hunyenyiwa et al. used the Lep^ob/ob^ mouse model of obesity to investigate angiogenesis following unilateral pneumonectomy, a critical aspect for successful regenerative lung growth. The authors showed that unilateral pneumonectomy inhibited the pro-angiogenic factor VEGF and its receptor VEGFR2 in an adiponectin dependent fashion. The use of adiponectin agonist aided in vascular and alveolar regeneration, suggesting potential applications for lung regeneration in obese individuals. Katsuki et al. discussed the role of PCSK9 in macrophage activation as a contributor towards the protective role of PCSK9 inhibition in the reduction of adverse cardiovascular outcomes in patients. The authors specifically highlighted the potential for PCSK9 to target receptors other than the classical target LDLR as well as how macrophage activation can be protected in both a lipid-dependent and lipid-independent fashion. Dritsoula et al. reviewed the role of secreted LRG1 as a vasculopathic molecule with the ability to disrupt angiogenesis and thereby impede vascular stability. This review described how LRG1 uses both canonical and non-canonical TGFβ signaling to affect the vasculature in a wide range of pathophysiological conditions. Chavkin et al. used a lineage tracing model of endothelial cells in mice fed a high fat/high sucrose diet as well as human adipose endothelial cells to investigate the presence of endothelial-mesenchymal transition (EndoMT) in obesity. The authors found a time-dependent increase in EndoMT in subcutaneous adipose tissue as well as increased expression of EndoMT gene markers when human adipose endothelial cells were cultured in the presence of pro-inflammatory stimulus. Their findings suggest that chronic obesity promotes EndoMT, which could lead to adipose tissue dysfunction.

## Novel imaging approaches

The second theme focuses on emerging trends in imaging which has enhanced the field of vascular biology research. Ning et al. described their own development of novel imaging modalities to look deeper into lower extremity peripheral artery disease. This case report showcases the use of near-infrared imaging (NIR-II 900-1880nm wavelength), optical coherence tomography angiography and laser speckle flowgraphy to show poor tissue perfusion in a patient with peripheral arterial disease suggesting the utility of these imaging modalities for the diagnoses of disease. Wayne et al. analyzed a cohort of 123 individuals with perfusion PET and coronary angiography with no obstructive coronary artery disease. The authors compared the Thrombolysis in Myocardial Infarction frame count (angiography-based method) and myocardial blood flow reserve (PET-based method) and found an inverse relationship between these parameters which in turn could be used to identify patients with coronary microvascular disease.

## COVID-19 pandemic

The third area was reflective of the emerging landscape of COVID-19 related vascular biology research. Iwata et al. used a machine learning based drug repurposing approach to identify FDA approved compounds with the ability to reduce surface expression of SARS-CoV-2 entry receptor, NRP1 specifically in macrophages. The authors validated that the top predicted compounds indeed reduce NRP1 surface expression in both cell lines and primary derived human macrophages. These findings have important implications for using drug repurposing-based approaches to rapidly identify agents to help prevent infection in niche subpopulations. Hatch et al. developed a vascularized micro-organ 3D microphysiological system consisting of endothelial cells and stromal cells to model SARS-CoV-2 infection using a pseudotyped GFP expressing lentivirus. This system was deployed to show infection with the virus could disrupt the vasculature by promoting inflammation and endothelial activation. Gao et al. developed a mouse model expressing human ACE2 to characterize COVID-19 pathogenicity in an *in vivo* setting. They concluded that direct infection of endothelial cells does not occur in this system and does not indeed contribute to vascular abnormalities. These findings suggest that COVID-19 associated vascular dysfunction can be attributed to inflammatory responses triggered by the viral infection.

## Data science

Four manuscripts represent the fourth theme, leveraging big data to find new trends in vascular biology. Husein et al. investigated three databases from 2008 to 2020 to calculate the impact of methamphetamine use in patients with pulmonary arterial hypertension. Similarly, Ali et al. investigated the National Inpatient Sample database from 2008 to 2020 to identify patients hospitalized with peripheral arterial disease. The use of ICD diagnoses facilitates comparison of sufficiently large real-world datasets to draw conclusions about the prevalence of disease and its associated factors. Dong et al. performed a mendelian randomization analysis on 1,400 serum metabolites in patients with peripheral arterial disease. Using this approach, the authors identified serum metabolites that showed a positive and others that showed a negative association with peripheral arterial disease diagnosis. Qin et al. performed a retrospective analysis of patients undergoing carotid stenosis from January 2018 to January 2022 in their department of endovascular surgery. Their investigation of 227 patients showed that several clinical parameters had independent capacity to predict asymptomatic carotid artery stenosis which could be used to help identify patients at risk.

## Summary and future perspectives

This collection highlights the interdisciplinary advances shaping the future of vascular biology. Through the exploration of cardio- and vascular-metabolic dysfunction, advanced imaging modalities, vascular implication of the COVID-19 pandemic and the integration of data science this collection underscores the complexity and evolving nature of vascular health. The insights presented not only deepen our understanding of disease but also point to innovative diagnostic and therapeutic strategies. This research topic shows how multiple different approaches can work together to provide a more comprehensive insight into disease progression. Such an interdisciplinary approach could rapidly advance our understanding of the disease and help speed up development of effective therapies. Continued research in these domains is essential for translating emerging knowledge into meaningful clinical outcomes.
